# *Cinnamomum* sp. and *Pelargonium odoratissimum* as the Main Contributors to the Antibacterial Activity of the Medicinal Drink Horchata: A Study Based on the Antibacterial and Chemical Analysis of 21 Plants

**DOI:** 10.3390/molecules28020693

**Published:** 2023-01-10

**Authors:** Paulina Fernandez-Soto, Diana Celi, Eduardo Tejera, José Miguel Alvarez-Suarez, António Machado

**Affiliations:** 1 Facultad de Ciencias de la Salud, Carrera de Enfermería, Grupo de Bio-Quimioinformática, Universidad de Las Américas (UDLA), Quito 170125, Ecuador; 2 Facultad de Ingeniería y Ciencias Aplicadas, Carrera de Ingeniería Agroindustrial, Universidad de Las Américas (UDLA), Quito 170125, Ecuador; 3 Facultad de Ingeniería y Ciencias Agropecuarias Aplicadas, Grupo de Bio-Quimioinformática, Universidad de Las Américas (UDLA), Quito 170125, Ecuador; 4 Colegio de Ciencias e Ingenierías, Departamento de Ingeniería en Alimentos, Universidad San Francisco de Quito (USFQ), Quito 170901, Ecuador; 5 Colegio de Ciencias Biológicas y Ambientales (COCIBA), Instituto de Microbiología, Laboratorio de Bacteriología, Universidad San Francisco de Quito (USFQ), Quito 170901, Ecuador

**Keywords:** horchata, *Cinnamomum* sp., *Pelargonium odoratissimum*, antibacterial activity, biofilm, natural products

## Abstract

Horchata, a herbal infusion drink from Ecuador containing a mixture of medicinal plants, has been reported to exhibit anti-inflammatory, analgesic, diuretic, and antioxidant activity. The antibacterial activity of each of the plants contained in the horchata mixture has not been fully evaluated. Thus, in this study, we analysed the antibacterial activity of 21 plants used in horchata, collected from the Ecuadorian Andes region, against bacterial strains of clinical importance. The methanolic extract of *Cinnamomum* sp. showed minimal inhibitory concentration (MIC) values of 250 µg/mL against *Staphylococcus aureus* ATCC25923 and Methicillin-resistant *S. aureus* (MRSA), while *Pelargonium odoratissimum* exhibited a MIC value of 500 µg/mL towards *S. aureus* ATCC25923. The high-performance liquid chromatography-diode array detector-tandem mass spectrometry (HPLC-DAD-MS/MS) analyses identified in *Cinnamomum* sp. epicatechin tannins, cinnamaldehyde, and prehelminthosporol molecules, whereas in *P. odoratissimum*, gallocatechin and epigallocatechin tannins, some flavonoids, and gallic acid and derivatives were identified. Finally, *Cinnamomum* sp. and *P. odoratissimum* showed partial inhibition of biofilm formation of *S. aureus* ATCC25923 and MRSA. Overall, our findings revealed which of the plants used in horchata are responsible for the antibacterial activity attributed to this herbal drink and exhibit the potential for *Cinnamomum* sp. and *P. odoratissimum* secondary metabolites to be explored as scaffolds in drug development.

## 1. Introduction

Natural products have been used since ancient times for the treatment of diseases and illnesses [1]. They comprise a large portion of current-day pharmaceutical agents and offer an alternative for identifying new compounds with novel chemical scaffolds [2,3,4]. The use of plants in traditional medicine is described in the literature of early civilizations in China, India, and the Near East, dating back over five millennia [2]. Plants are a great source of bioactive compounds (secondary metabolites), most of which are phenols or their oxygen-substituted derivatives, such as flavonoids, alkaloids, lignans, stilbenes, tannins, terpenes, polyphenolics, quinones, and coumarins, among others [2,5,6]. Secondary metabolites are found in different parts of plants, including seeds, roots, stems, leaves, barks, and fruits [5]. Several studies have described the potential of secondary metabolites from plants to exert antioxidant activity [7], antimicrobial activity against susceptible and resistant bacteria [2,8,9], as well as biofilm and quorum sensing (QS) disruptor activity [8].

Tropical countries, such as Ecuador, contain a vast variety of native and adapted flora with the possibility of being exploited for drug discovery and development. The terms herbs and species vary depending on the part of the plant used and are commonly employed in traditional medicine and for culinary purposes [10]. A herbal infusion named horchata is a popular drink in the Loja Province, situated in southern Ecuador, and contains a mixture of 16–32 medicinal plants [11]. The number of medicinal plants in the horchata infusion varies depending on availability, and even though the herbs are sold with the same species name, they differ according to national regions. Furthermore, the amount of each plant used for horchata preparation is not currently known. In Ecuador, horchata is commonly sold at the local markets as either a ready-to-drink infusion or as a bunch of dried plants mixed randomly by each seller [11]. Among the most frequently used species in the horchata drink are: *Amaranthus hybridus* (Ataco), *Aloysia triphylla* (Cedrón), *Althaea officinalis*, *Adiantum concinnum* (Culantrillo), *Aerva sanguinolenta* (Escancel), *Alcea rosea*, *Borago officinalis* (Borraja), *Cymbopogon citratus* (Hierba luisa), *Cinnamomum* sp. (Canela), *Citrus x junos or Citrus x aurantium* (Hoja de naranja), *Dianthus caryophyllus*, *Equisetum bogotense* (Cola de caballo), *Foeniculum vulgare*, *Fuchsia hybrida*, *Fuchsia loxensis* (Pena pena), *Malva* sp., (Malva esencia), *Matricaria chamomilla* (Manzanilla), *Mentha x piperita* (Menta) or *Menta spicata*, *Melissa officinalis* (Toronjil), *Origanum vulgare* (Oregano), *Ocimum basilicum* (Albahaca blanca), *Ocimum tenuiflorum* (Albahaca Negra), *Plantago major* (Llantén), *Pelargonium graveolens* or *P. odoratissimum* (Malva olorosa), *Peperomia inaequalifolia* (Congona), *Pimpinella aromatica* (Pimpinela), *Stevia rebaudiana* (Stevia), *Triumfetta semitriloba* (Cadillo), and *Viola odorata* (Violeta) [11,12].

The horchata mixture exhibits anti-inflammatory, analgesic, diuretic, and antioxidant activity [11,12,13,14]. Our group identified that the hydroalcoholic extract (methanol-water, 80:20 *v*/*v*) of the horchata mixture contains a wide range of phenolic compounds, the most prominent being the flavones, flavonols (e.g., quercetin glycosides and apigenin glycosides), and chlorogenic acids (such as caffeoylquinic and dicaffeoylquinic acids) [12]; compounds found in different medicinal plants and with known antimicrobial properties [1,2,15]. However, previous studies did not analyse the plants separately but as a group. Therefore, the specific contribution of each plant to the overall effect remains unknown. Other studies have reported antimicrobial activity by some of the plants used in horchata; for instance, *Amaranthus* sp., *Aloysia triphylla*, *Cinnamomum* sp., *Matricaria chamomilla*, *Mentha x piperita*, *Melissa officinalis*, *Ocimum basilicum*, and *Ocimum tenuiflorum* [2,9,16]. It is important to mention that none of these studies are from the Ecuadorian region. In addition, some of the plants have been used as part of a mixture with other well-known medicinal plants to augment their activity; for instance, *Ocimum basilicum*, *Citrus x aurantium*, and *Origanum vulgare* used in combination with *Aaronsohnia factorovskyi*, a carminative agent [8]. However, there is no evidence of antibacterial activity from either the horchata mixture or the contribution of each plant contained in the horchata, despite the antibacterial activity attributed to this medicinal drink, especially from plants collected from the Ecuadorian Andes region [17]. Moreover, information on the antibacterial activity of horchata plants towards pathogens described as having priority status by the WHO remains scarce [18]. In this context, we aimed (i) to evaluate the antibacterial activity of 21 medicinal plants used in horchata against susceptible and resistant bacterial strains of clinical importance, (ii) to chemically characterise the plants with the best antibacterial activity, and (iii) to assess the biofilm inhibition and/or eradication activity of plants showing planktonic antibacterial activity.

## 2. Results

### 2.1. Total Phenolic Content, Total Flavonoid Content, and Total Antioxidant Capacity

In this study, 21 crude plant extracts were analysed for their total phenolic content (TPC), total flavonoid content (TFC), and total antioxidant capacity (TAC). The TPC, TFC, and TAC of each crude plant extract are shown in Table 1. The TPC results varied widely from 28.71 to 382.1 mg GAE per g DE. The 21 extracts were divided into three groups according to their TPC values. The first group, with TPC values less than 75 mg GAE per g DE, included *Amaranthus hybridus*, *Cymbopogon citratus*, *Matricaria chamomilla*, *Althaea officinalis*, *Plantago major*, *Ocimum tenuiflorum*, *Borago officinalis*, *Aerva sanguinolenta*, *Mentha x piperita*, *Fuchsia loxensis*, and *Citrus x aurantium*. The second group, with TPC values between 75 and 200 mg GAE per g DE, included *Ocimum basilicum*, *Origanum vulgare*, *Aloysia triphylla*, *Adiantum concinnum*, and *Stevia rebaudiana*. Finally, the third group, with TPC values higher than 200 mg GAE per g DE, included *Cinnamomum* sp., *Melissa officinalis*, *Pelargonium odoratissimum*, and *Equisetum bogotense* (Table 1). The TFC ranged between 5.9 and 256.4 mg CE per g DE. The highest values regarding TFC were found for *Melisa officinalis* and *Cinnamomum* sp. (Table 1). However, it is important to notice not only the total content but also the relative content with respect to the TPC. A significant linear correlation was found (R^2^ = 0.557, *p*-value < 0.05) between TPC and TFC. However, this correlation is considerably increased (R^2^ = 0.856, *p*-value < 0.01) by removing two of the plants, *Pelargonium odoratissimum* and *Equisetum bogotense* (which were quite different from the linear trend). These two plants had higher values of TPC but the lowest TFC, suggesting that the values obtained from TPC in those cases are not related to flavonoids but mainly to a different molecular space with a reduction capacity or a different polyphenol family.

The TAC was determined using the 2,2-diphenyl-1-picrylhydrazyl (DPPH) free radical method and the ferric reducing antioxidant power (FRAP) assays (Table 1). DPPH ranged from 79.9 to 2894.3 µmol TEq per g DE, which represents a 36-fold variation. The 21 crude plant extracts were divided into three groups according to DPPH values. The first group is comprised of extracts with low values of DPPH < 400 µmol TEq per g DE (*n* = 11), the second group with medium values of 400–1000 µmol TEq per g DE (*n* = 7), and the third group of extracts with high values of >1000 µmol TEq per g DE (*n* = 3). FRAP ranged from 71.0 to 1582.4 µmol TEq per g DE. The trend for the FRAP of the 21 crude plant extracts did not vary markedly from their DPPH. A similar classification to the DPPH is presented for the plant extracts according to FRAP values. The first group is comprised of extracts with low values of FRAP <400 µmol TEq per g DE (*n* = 12), the second group with medium values of 400–800 µmol TEq per g DE (*n* = 6), and the third group of extracts with high values of >1000 µmol TEq per g DE (*n* = 3). Overall, *Melissa officinalis*, *Cinnamomum* sp., and *P. odoratissimum* showed high values of DPPH and FRAP. A very significant linear correlation was found between TPC and FRAP (R^2^ = 0.884, *p*-value < 0.01), and TPC and DPPH (R^2^ = 0.890, *p*-value < 0.01). These results indicate that TPC is a key factor in determining the TAC of these extracts. This relationship is fulfilled for all extracts except for *Equisetum bogotense*, which showed high TPC, but low antioxidant capacity.

### 2.2. Antibacterial Activity of Crude Extracts

Likewise, we evaluated the antibacterial activity of the 21 plants using the well diffusion method, and further selection was performed with a microdilution assay. The 21 crude methanol extracts were initially screened at a concentration of 50 mg/mL against a set of eight susceptible bacterial strains (Table 2) and six resistant strains (Table 3), and their potencies were assessed qualitatively and quantitatively by the presence or absence of inhibition zones and zone diameters. The 50 mg/mL concentration was set for the initial screening to overcome any low diffusion of the crude extracts in the agar and avoid false negatives. The results showed that 12 crude methanol extracts had an inhibition effect (zone diameters between 2 and 17 mm) on the growth of the susceptible strains *E. faecalis*, *S. aureus*, *K. pneumoniae*, *A. baumannii*, *E. cloacae*, and *E. coli* (Table 2), of which three extracts showed inhibition on the growth of the MRSA 333 clinical strain (Table 3). Only the crude extracts that showed inhibitory effects on the susceptible strains (Table 2) were further assessed for their inhibitory effect on the resistant strains available in our laboratory (Table 3). It is noteworthy that no inhibitory activity on the growth of *P. aeruginosa* by any of the 21 crude extracts tested was observed.

To further validate the inhibitory activity of the crude extracts selected by the well diffusion assay, the minimum inhibitory concentration (MIC) and minimum bactericidal concentration (MBC) were determined. For this, a maximum starting concentration of the crude extracts was set at 1000 µg/mL. The in vitro susceptibility tests showed that *Cinnamomum* sp. and *P. odoratissimum* had antibacterial effects against the gram-positive bacteria *S. aureus* with MIC values of 250 and 500 µg/mL, respectively (Table 4). In addition, *Cinnamomum* sp. showed inhibition on the growth of the MRSA 333 with a MIC value of 250 µg/mL. Overall, *Cinnamomum* sp. methanol extracts showed the best inhibitory activities over the growth of both *S. aureus* and MRSA 333 strains. It is noted that some of the crude extracts with antibacterial activity detected by the well diffusion assay with zone diameters <11 mm (Table 2) showed inhibitory effects at MIC values >1000 µg/mL; however, inhibitory activities with MIC values >1000 µg/mL were considered in our study as ineffective. The negative control methanol/water (80:20, *v*/*v*) used as a solvent control for all the antimicrobial activity analyses, showed no visible inhibitory effect on bacterial growth. MIC values of the positive control ciprofloxacin were, as recommended, within plus or minus one or two-fold dilution of the expected MIC [19,20].

The extract *Cinnamomum* sp. and *P. odoratissimum* showed MBC values against *S. aureus* of 250 and 1000 µg/mL, respectively, while the MBC value against the MRSA 333 strain by *Cinnamomum* sp. was 500 µg/mL (Table 4). The MBC/MIC ratio indicates the type of antimicrobial activity of a drug; thus, a value lower or equal to four indicates bactericidal activity, and a value higher than four indicates bacteriostatic activity [21]. *Cinnamomum* sp. and *P. odoratissimum* have bactericidal activities with an MBC/MIC ratio lower than four; therefore, acting as ‘traditional antibiotics’ by their capacity to kill bacteria directly.

### 2.3. Biofilm Inhibition and Eradication Assays

**Figure 1:**
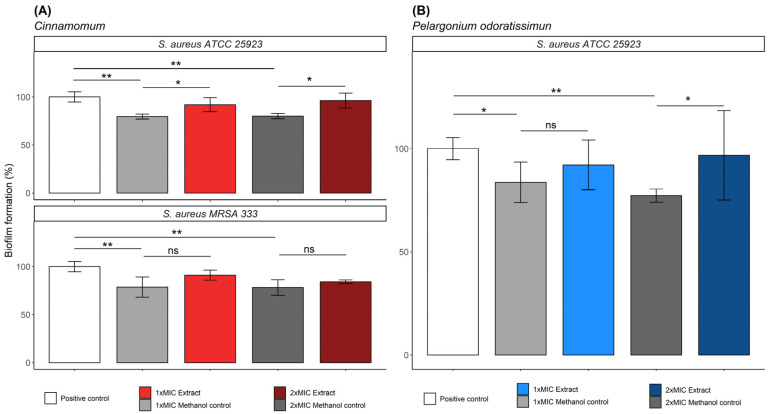
Representative illustration of the biofilm inhibition assays by extract plants used in the herbal infusion horchata. (**A**) Evaluation of the antibiofilm activity of *Cinnamomum* sp. extract against *S. aureus* and MRSA 333. (**B**) Evaluation of the antibiofilm activity of *Pelargonium odoratissimum* extract against *S. aureus*. All statistical analyses (*p*-values) were analysed using a nonparametric Mann–Whitney test (95% confidence interval) for comparison between biofilm formation values, where: * *p*-values < 0.05; ** *p*-values < 0.01; and ns: nonsignificant *p*-value.

Following the MIC and MBC of the plant extracts, *Cinnamomum* sp. and *P. odoratissimum* extracts were selected to further analyse their ability to inhibit and eradicate biofilms against *S. aureus* and MRSA 333. *Cinnamomum* sp. extract showed different degrees of inhibition for *S. aureus* and MRSA 333, being more efficient against MRSA 333, with a biofilm inhibition of approximately 15–20% compared to the positive control (bacteria growth in culture media only) (Figure 1A; Suppl. Table S1).

In addition, the *P. odoratissimum* extract demonstrated a similar biofilm inhibition against *S. aureus* (Figure 1B). Interestingly, we found no significant effect on biofilm inhibition when increasing the MIC values of *Cinnamomum* sp. or *P. odoratissimum* extracts up to 2× MIC concentration, evidencing a better biofilm inhibition at 1× MIC concentration against both *S. aureus* and MRSA 333 (Suppl. Table S1). All the methanol/water (80:20, *v*/*v*) negative controls revealed statistical differences when compared with the positive control, indicating a biofilm inhibition by itself, and also differing from the biofilm inhibition values obtained by these two plant extracts (Figure 1). Overall, the plant extracts demonstrated their abilities to partially inhibit biofilm formation from *S. aureus* and MRSA 333, independent of the presence of methanol.

**Figure 2:**
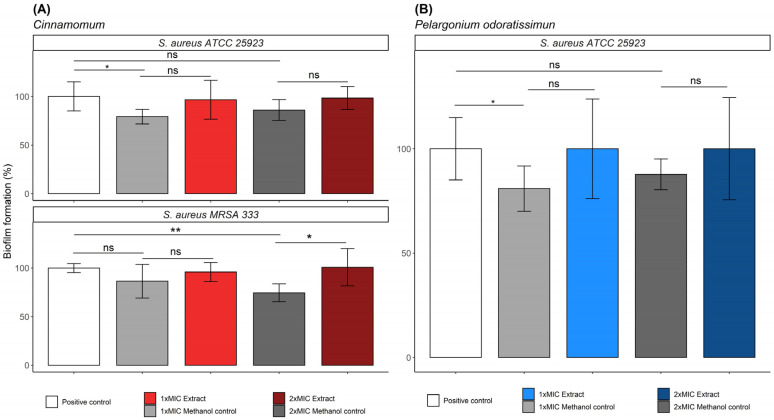
Representative illustration of the biofilm eradication assays using extracts from plants used in the herbal infusion horchata. (**A**) Evaluation of the antibiofilm activity of *Cinnamomum* sp. extract against *S. aureus* and MRSA 333. (**B**) Evaluation of the antibiofilm activity of *Pelargonium odoratissimum* extract against *S. aureus*. All statistical analyses (*p*-values) were analysed using a nonparametric Mann–Whitney test (95% confidence interval) for comparison between biofilm formation values, where: * *p*-values < 0.05; ** *p*-values < 0.01; and ns: nonsignificant *p*-value.

Furthermore, when applying the same plant extracts against 24 h biofilms of *S. aureus* and MRSA 333 strains, a slight decrease in biofilm reduction was observed (≈4%) only by *Cinnamomum* sp. extract at 1× MIC concentration (Figure 2A; Suppl. Table S2), and no biofilm reduction in *S. aureus* by *P. odoratissimum* extract was seen in both 1× and 2× MIC concentrations (Figure 2B; Supplementary Table S2). None of the negative controls revealed statistical differences with the biofilm eradication values obtained by these two plant extracts, except for *Cinnamomum* sp. at 2× MIC concentration against MRSA 333 (Figure 2A). Altogether, our data showed that no significant reduction was obtained in 24 h biofilms of S. aureus and MRSA 333 by *Cinnamomum* sp. and *P. odoratissimum* extracts.

### 2.4. Identification of Chemical Compounds in Cinnamomum sp. and Pelargonium odoratissimum (L.) L’Hér Hydroalcoholic Extracts

*Cinnamomum* sp. and *P. odoratissimum* were identified as the plants with the best antibacterial activity among the 21 plants tested. Consequently, we analysed the chemical composition of these two plants by HPLC-DAD-MS/MS, and the tentative identification is presented in Table 5 and Table 6. We first analysed the results in positive ionisation mode, adding those identifications that were found in negative ionisation but not in positive.

In ID1, several molecules were detected (Table 5). The mass at 180 m/z with product ions at 121 and 165 m/z was consistent with candicine, as previously described [22]. However, the same mass and rupture pattern also appeared in ID2 (RT = 3.06) which we suggested as a candicine isomer. The parent mass at 381 m/z could be assigned to the oxidised disaccharide product [2-hexose+O+Na]+ with further product ions at 219 [hexose+O+Na]+ due to the loss of one hexose residue from the nonreducing end (loss of 162 uma from 381–219 m/z) [23].

The parent mass of 1153 m/z was detected at several retention times (IDs 5, 6, and 7 in Table 5). The parent ion at 1153 m/z (ID5) was fragmented at 1001 m/z (loss of 152 uma corresponding with retro-Diels–Alder fragmentation reactions), 863 m/z (loss of 290, catechin unit), and 985 m/z. On the other hand, in the ID6, the rupture changed to 865 m/z (loss of 288, corresponding with the quinone methide reaction). Similar patterns have been reported in positive ionisation mode for (Epi)catechin tetramers as EC-A-EC-EC-EC and EC-EC-A-EC-EC, respectively, based specifically on the order of elution [24]. This identification is consistent with the loss of one of the monomers producing an ion at 865 m/z (ID8), which ruptures at 533 m/z; 713 m/z has previously been identified as A-type procyanidin protonated trimer (EC-A-EC-EC) [24,25] and also in positive ionisation mode. We cannot reject the possibility that some of the 865 m/z was a product of 1443 m/z [M+H-288–290]+, indicating a penta-polymer of (epi)catechin, and 1153 m/z was a product ion of 1443 m/z [M+H-290]+. Similarly, other authors have reported the 1443 m/z as a double-charged ion, pointing towards a hexamer of catechin [26]. Moreover, the parent mass at 579 m/z (ID6) produced an ion at 427 m/z, a loss of 152 uma (corresponding with retro-Diels–Alder fragmentation reactions), and 291 m/z, corresponding with the loss of one of the catechins. This mass was tentatively identified as a catechin dimer (Proanthocyanidin B2) and some isomer at ID9.

The rupture of 314 m/z (ID6, Table 5) in 297 m/z has been reported previously [24] as norboldine (laurolitsine) in other *Cinnamomum* species. However, 265 m/z instead of 269 m/z should be present. The loss of 17 uma (314–297 m/z) could be associated with an amine loss and a further loss of the hydroxyl group as water (297–269 m/z), leading us to identify it as a more hydrophilic derivate of coclaurine. In the work of Lin et al. [27], a similar rupture at 314 m/z and retention time earlier than coclaurine was identified as lotusine. On the other hand, another 314 m/z parent mass was noticed at ID8 (Table 5) with a similar rupture as coclaurine and a primary fragment at 283 and 169 m/z. This rupture has been previously identified as 4′-methyl-N-methylcoclaurine [27].

In ID10 (Table 5), we also found a parent mass of 149 m/z, which also appeared in RT = 19.45 (ID12). In both cases, the majoritarian rupture was at 149 m/z. However, product ions with low intensities were observed at 121 and 131 m/z. This rupture was previously identified as 2-hydroxycinnamaldehyde and cinnamic acid [28]. Based on the elution order previously reported [24,28], using a similar chromatographic mobile and stationary phase, we identified the first to be the 2-hydroxycinnamaldehyde and the later one to be the cinnamic acid. Moreover, at RT = 22.70 (ID15, Table 5), we detected an intense ion at 163 m/z. This ion was poorly fragmented, but the following ions were detected with low intensity: 135, 145, and 104 m/z. These ions and the 163 m/z correspond with 4-methoxycinnamaldehyde or 2-methoxycinnamaldehyde. These aldehydes are frequently and abundantly found in the *Cinnamomum* species [28]. We expected the characteristic mass of 55 m/z; however, we think that higher collision energy is needed. The other source of 163 m/z is derived from 195 m/z (ID16). The consecutive loss of two oxygens explains the change to 163 m/z from 195 m/z. Therefore, a minor methoxycinnamaldehyde derivate was indicated as a possible additional identification.

**Table 5:** Metabolites identified in *Cinnamomum* sp. by HPLC-ESI-MS/MS in positive and negative ionisation mode.

Positive Ionisation						
ID	RT (min)	UV	Parent Mass (m/z)	Main MS/MS Products (m/z)	Tentative Identification	References
1	1.16	250, 266	203 ([M+Na]^+^)	185 (100), 113 (90), 143 (50), 172 (20)	Fructopyranose	[29]
	1.21	250, 266	219 ([M+K]^+^)	201 (100), 89 (45), 210 (10), 72 (10)	Fructose	[30]
	1.27	250, 266	381 ([2-hexose+O+Na]^+^)	363 (100), 219 (30), 297 (25), 201 (15), 345 (15)	Disaccharide	[23]
	1.27	250, 266	266 ([M+H]^+^)	248 (100), 230 (5)	UI	
	1.27	250, 266	180 ([M+H]^+^)	121 (100), 165 (40), 60 (30)	Candicine	[22]
2	3.06	222, 274	180 ([M+H]^+^)	121 (100), 165 (40), 60 (30)	Candicine isomer	[22], see comments
3	6.42	214, 278	223 ([M+H]^+^)	207 (100), 225 (15)	4-(2,3-Dihydro-1,4-benzodioxin-6-yl)butanoic acid	86.8%
4	6.47	214, 278	208 ([M+H]^+^)	131 (100), 149 (50), 103 (10)	UI	
5	9.87	214, 278	1153 ([M+H]^+^)	1001 (100), 863 (50), 985 (35)	A type (Epi)catechin tetramer (EC-EC-EC-A-EC-EC)	[24], see comments
6	11.56	214, 278	314 ([M+H]^+^)	297 (100), 298 (98), 269 (60), 175 (10), 237 (5)	Lotusine	[27], see comments
	11.69	218, 278	1153 ([M+H]^+^)	865 (100), 1001 (90), 983 (45), 985 (40)	A Type procyanidin pentamer	[24], see comments
	11.83	218, 278	579 ([M+H]^+^)	427 (100), 409 (80), 291 (70), 301 (25), 247 (20), 561 (5)	A type of proanthocyanidin B	[24,25], see comments
	12.24	214, 278	286 ([M+H]^+^)	269 (100), 175 (10), 107 (5), 237 (5)	Coclaurine	[31], see comments
7	12.43	230, 278	300 ([M+H]^+^)	269 (100), 175 (15), 107 (5), 237 (5)	N-methylcoclaurine	[31], see comments
	12.43	230, 278	291 ([M+H]^+^)	123 (100), 139 (85), 165 (45), 273 (35), 151 (30)	Epicatechin	96.5%
	12.83	218, 278	1153 ([M+H]^+^)	865 (100), 1001 (90), 983 (45), 985 (40)	A type of procyanidin pentamer, isomer	[24], see comments
8	12.45–12.97	230, 278	865 ([M+H]^+^)	533 (100), 713 (60), 828 (35), 695 (30)	A type procyanidin trimer (EC-A-EC-EC)	[24,25], see comments
	13.00	218, 278	314 ([M+H]^+^)	283 (100), 269 (40), 299 (25), 107 (20)	4′-Methyl-N-methylcoclaurine	[27], see comments
9	13.38	214, 278	330 ([M+H]^+^)	192 (100), 299 (10)	Reticuline	[32,33]
	15.04	214, 278	579 ([M+H]^+^)	409 (100), 427 (75), 561 (50), 291 (50), 453 (50)	A type of procyanidin B isomer	see comments
10	16.88	214, 278	149 ([M+H]^+^)	149 (100)	2-Hydroxycinnamaldehyde	[28], see comments
11	19.24	214, 278	693 ([M+H]^+^)	541 (100), 523 (80), 389 (10)	UI	
12	19.45	214, 274	149 ([M+H]^+^)	149 (100)	Cinnamic acid	[28], see comments
13	19.63	214, 278	423 ([M+H]^+^)	405 (100), 387 (59), 271 (50)	Mangiferin	[34]
14	20.14	214, 278	237 ([M+H]^+^)	219 (100), 201 (60), 191 (60), 180 (40)	Prehelminthosporol	[35]
15	22.70	214, 286, 388	195 ([M+H]^+^)	163 (100), 154 (10), 167 (8), 180 (8), 135 (5)	Methoxycinnamaldehyde derivate	[28], see comments
16	22.89	234, 290, 338	163 ([M+H]^+^)	163 (100)	Methoxycinnamaldehyde (see notes)	[28], see comments
Negative ionisation						
1	1.25	230,278	191 ([M-H]^−^)	111 (100), 173 (50), 85(25), 127 (20)	Quinic acid	87.8%
2	1.32	242, 278	195 ([M-H]^−^)	129 (100), 177 (80), 159 (40), 141 (5), 111 (5)	Galactonic acid	82.0%
3	13.50	226, 286	206 ([M-H]^−^)	164 (100), 147 (15)	N-acetyl-L-Phenylalanine	97.9%

Notes: unidentified compound (UI). The percentage indicates the score reported in the MzCloud database.

The precursor mass at 293 m/z (ID 1, Table 6) was not identified. However, similar ruptures of 293–275 m/z and ions at 234 m/z (instead of 233 m/z) have been identified as fusarochromanone (MassBank database). The parent mass of 611 m/z (ID3, Table 6) also appeared at other retention times: RT = 10.28 (ID5, Table 6), and RT = 14.91 (at the same level as ID14) with the same rupture pattern. Other masses were found at those times, such as 915 m/z and 1219 m/z. Even when it was not entirely possible to confirm the rupture patterns of those masses according to the retention times, these masses have been associated with 611 m/z, indicating several degrees of polymerization (611-dimer, 915-trimer, and 1219-tetramer of gallocatechin) [36]. The mass at 449 m/z also appeared in the RT= 13.85 min (ID 11, Table 6), with the same rupture, indicating a possible isomer of luteolin glucoside.

**Table 6:** Metabolites identified in *Pelargonium odoratissimum* (L.) L’Hér by HPLC-ESI-MS/MS in positive and negative ionisation modes.

Positive Ionisation						
ID	RT (min)	UV	Parent Mass (m/z)	Main MS/MS Products (m/z)	Tentative Identification	References
1	1.06–1.55	250, 290	175 ([M+H]^+^)	157 (100), 175 (50), 130 (60), 116 (45), 60 (35)	L-(+) arginine	96.1%
			219 ([M+H]^+^)	201 (100), 159 (25), 173 (20), 90 (10)	5-Acetyl-2-(1-hydroxy-1-methylethyl)benzofuran	[37]
		222	182 ([M+H]^+^)	165 (100), 136 (25)	L-tyrosine	87.7%
			293 ([M+H]^+^)	275 (100), 233 (40), 209 (20), 203 (15), 118 (10)	UI	see comments
2	1.62	214, 270	371 ([M+H]^+^)	283 (100), 353 (70)	UI	
	2.50	214, 266	166 ([M+H]^+^)	166 (100), 120 (30)	L-phenylalanine	93.9%
3	4.94	214, 270	611 ([M+H]^+^)	443 (100), 425 (15),485 (5)	Polymer of gallocatechin	[36], see comments
	7.39		307 ([M+H]^+^)	139 (100), 151 (80), 289 (80)	Epigallocatechin	93.7%
4	8.49	218, 278	205 ([M+H]^+^)	188 (100), 159 (5)	L-Tryptophan	95.9%
5	10.28	218, 278	611 ([M+H]^+^)	425 (100), 443 (80), 317 (35), 287 (30), 467 (30), 485 (30), 593 (30)	Polymer of gallocatechin	[36], see comments
6	11.57	218, 278	389 ([M+H]^+^)	209 (100), 227 (35), 191 (20), 371 (10), 173 (10)	4-[4-Hydroxy-2,6,6-trimethyl-3-[3,4,5-trihydroxy-6-(hydroxymethyl)oxan-2-yl]oxycyclohexen-1-yl]butan-2-one	81.6%
7	11.89	238, 282	690 ([M+H]^+^)	316 (100) 316->298 (100), 209 (30),227 (25),177 (5)	UI	
8	12.53	222, 274	783 ([M+H]^+^)	303 (100), 277 (40), 337 (30), 463 (20),765 (10)	UI	
9	12.69	230, 278	459 ([M+H]^+^)	289 (100), 151 (20), 139 (20)	Epigallocatechin gallate	95.0%
10	13.80	218, 266, 350	449 ([M+H]^+^)	431 (100), 383 (60), 353 (25), 329 (20)	Luteolin-8-glucoside	95.2%
11	13.85	226, 270, 350	449 ([M+H]^+^)	431 (100), 383 (60), 353 (25), 329 (20)	Luteolin glucoside isomer	see comments
12	13.87	218, 266, 346	348 ([M+H]^+^)	169 (100), 331 (20), 123 (20), 313 (15), 151 (10)	UI	see comments
	14.62	222, 266, 342	619 ([M-H_2_O+H]^+^)	449 (100), 237 (70), 261 (20), 279 (10), 431 (5)	1,2,6-tri-O-galloylglucose	88.8%
13	14.78	222, 266, 342	376 ([M+H]^+^)	197 (100), 179 (20), 358 (5)	UI	
	14.91		611 ([M+H]^+^)	303 (100), 465 (30)	Rutin	see comments
14	15.00	222, 266, 342	433 ([M+H]^+^)	415 (100), 367 (50), 271 (40), 397 (25), 337 (20), 313 (20), 379 (15)	Vitexin	92.8%
	15.00	318, 266, 346	435 ([M+H]^+^)	417 (100), 399 (25), 369 (20), 315 (15), 339 (10)	Naringenin-7-O-glucoside	84.5%, see comments
	15.24	222, 266, 342	465 ([M+H]^+^)	303 (100), 447 (10)	Quercetin hexoside	see comments
15	15.38	218, 278	334 ([M+H]^+^)	299 (100), 317 (25), 177 (5)	UI	
16	15.93	214, 278	595 ([M+H]^+^)	287 (100), 449 (30), 576 (5) 449-> 287 (100), 431 (90), 383 (45)	Kaempferol 3-neohesperidoside	92.4%, see comments
17	16.63	214, 278	449 ([M+H]^+^)	287 (100), 431 (90), 383 (50)	Trifolin	96.1%, see comments
18	17.71	214, 286	481 ([M+H]^+^)	303 (100), 285 (90), 301 (75), 463 (20), 319 (20)	Myricetin 3-galactoside	see comments
19	18.61	214, 282	481 ([M+H]^+^)	301 (100), 319 (50), 283 (45), 463 (20)	UI	
20	19.50	214, 274	303 ([M+H]^+^)	257 (100), 285 (35), 165 (5), 229 (5)	Quercetin	see comments
Negative ionisation						
21	1.06–1.55	250, 290, 314	169 ([M-H]^−^)	169 (100), 125 (20)	Gallic acid	87.1%
			331 ([M-H]^−^)	169 (100), 271 (80), 211 (40), 125 (10)	Galloylglucose	[38]
22	12.71	218, 278	951 ([M-H]^−^)	907 (100), 934 (80)	Geraniin	Refs. [39,40,41], see comments
23	14.07	214, 270, 346	447 ([M-H]^−^)	327 (100), 357 (75), 429 (25)	2-(3,4-Dihydroxyphenyl)-5,7-dihydroxy-8-[(3R,4R,5R,6R)-3,4,5-trihydroxy-6-(hydroxymethyl)oxan-2-yl]chromen-4-one	84.0%
24	14.79	222, 278, 338	951 ([M-H]^−^)	934 (100), 933 (70)	Granatin B	Refs. [39,40,41], see comments
25	15.15	230, 270, 338	301 ([M-H]^−^)	257 (100), 185 (50)	Ellagic acid	[42]
26	15.34	222, 270, 338	433 ([M-H]^−^)	313 (100), 387 (30), 301 (25), 343 (10)	Hemiphloin	75.4%
27	17.50	214,274	429 ([M-H]^−^)	249 (100), 267 (30)	Formononetin-7-O-glucoside	[43]

Notes: unidentified compound (UI). The percentage indicates the score reported in the MzCloud database.

The parent mass of 348 m/z (ID12, Table 6) has been reported as the best hit (score 78.2) in MzCloud as the vanillic acid glucoside. This molecule has a molecular mass of 330. The loss of 162 uma (hexoxide) from 331 to 169 m/z was consistent with a glucoside derivate. Even so, the parent mass of 348 m/z did not correspond with the 331 mass expected for vanillic acid glucoside. Even when 151 and 313 m/z were consistent with the vanillic spectra in positive ionisation in the library, the retention time was too long for a molecule such as this one. Moreover, the intensity of the 449 m/z remained at the top of our spectrum, indicating that it is difficult to identify any other molecule in this chromatographic region. We found two main precursor masses at 376 m/z and 611 m/z (ID13, Table 6). The 611 m/z fragmented in 303 m/z was consistent with a loss of rutinoside and quercetin product ion. However, the rupture pattern of 376 m/z in 197 and 179 m/z was not identified (probably because of low energy). We tentatively identified this molecule as rutin. Similarly, the molecular ion at 346 m/z lost a hexoside moiety (162 uma) and was tentatively identified as quercetin hexoside. For parent mass 435 m/z (ID14), the best hit (score 84.5) was found in MzCloud for naringin. The molecular mass and rupture (also indicated in MzCloud) were consistent with naringenin-7-O-glucoside. This molecule explains better the 435 m/z [M+H]+. The compound at 619 m/z was identified in MzCloud as 1,2,6-tri-O-galloylglucose with a score of 88.8, but this compound has a molecular mass of 636, consequently, we proposed the adduct as [M-H2O+H] at 619 m/z.

In ID15 (Table 6), we found a molecular ion at 334 m/z with product ions at 299 and 317 m/z. This rupture pattern was not identified. The parent mass at 595 m/z (ID16) was identified in MzCloud (score 92.4) as kaempferol 3-(2G-rhamnosylrobinobioside), with a molecular mass of 740. However, we finally proposed the kaempferol 3-neohesperidoside, which has a similar spectrum but lost one of the sugar units and had a molecular mass of 594. In ID17 (Table 6), we found a molecular ion at 449 m/z. This rupture was identified as 5,7-Dihydroxy-2-(4-hydroxyphenyl)-4-oxo-4H-chromen-3-yl-6-O-(6-deoxyhexopyranosyl) hexopyranoside in MzCloud with a score of 96.1. However, this molecule had a molecular mass of 594, which is higher than our parent mass of 449 m/z. The rupture of 449–431 m/z was consistent with the loss of water, and the molecular ion at 431 m/z corresponded to kaempferol. Therefore, the MzCloud identification is correct but exceeded in a glucoside, and that is why we suggested trifolin (molecular mass of 448) instead of kaempferol. Two other molecules were identified in the positive ionisation mode: ID18 and ID20 (Table 6). The parent mass at 481, producing a rupture in 319 and 303 m/z, corresponds with the loss of the galactoside connected to myricetin with a further loss of water to form the ion at 285 m/z. The rupture pattern of the parent mass at 303 m/z was also consistent with quercetin. The parent mass at 481 m/z (ID18), with a primary rupture at 301 m/z instead of 303 m/z, was not identified.

In the negative ionisation mode, the parent mass at 951 m/z was present at two retention times, RT = 12.71 (ID22) and 14.79 (ID24). These masses have been described as polymers related to ellagic acid (that was identified in ID25), specifically geraniin and granatin B [39,40,41]. The analysis of both compounds was based on the mass rupture but mainly on taking the retention time and elusion order into consideration.

## 3. Materials and Methods

### 3.1. Plant Materials and Extract Preparation

For this study, a total of 21 plants were selected based on their frequent use in horchata drink preparation [11,12] and availability. Table 7 shows the common name, the scientific name, and the part of each plant traditionally used for the elaboration of horchata, which is also the part of the plant used for this study. Plants were identified and confirmed as explained in our earlier work by specialists from the Botanical Garden of Quito, Ecuador, using the reference samples deposited in the herbarium of this centre and those deposited from previous studies [12,13]. Whole and fresh plants (0.5 kg per specimen) were purchased during March 2022 in two of the most traditional markets in the Metropolitan District of Quito: (i) Santa Clara Market and (ii) Iñaquito Market. Fresh plants were deep frozen at −80 °C for 3 h, then freeze-dried in a lyophilizer, Büchi Lyovapor L-200 (BÜCHI Labortechnik, Flawil, Switzerland), for approximately 48 h. Once dried, plant samples were finely ground with liquid nitrogen in a laboratory porcelain mortar and stored at −20 °C until analysis. For extract preparation, 2 g of the ground sample from each of the 21 plants were extracted with 20 mL of methanol (Sigma-Aldrich, Burlington, MA, USA)-water solution (80:20, *v*/*v*), stirred, and left for 2 h in the dark at room temperature. The samples were centrifuged twice for 10 min at 1500 RPM and filtered through cellulose filter paper (0.2 µm; Whatman, Merck & Co., Inc., St. Louis, MO, USA). The supernatant was concentrated under vacuum using a Büchi Rotavapor R-210 set (BÜCHI Labortechnik, Flawil, Switzerland) at 18 °C in the water bath and the cooling water at 0 °C until most of the solvent was eliminated. Then, the concentrate was brought to dryness using a miVAC centrifugal concentrator (GeneVacTM, Thermo Fisher Scientific, Uppsala, Sweden) set at 30 °C for 5 h. Dry extracts were stored at −80 °C until use. Stock solutions were prepared by resuspending the dry residue in 1 mL of a methanol-water solution (80:20, *v*/*v*).

### 3.2. Total Phenolic Content, Total Flavonoid Content, and Total Antioxidant Capacity Assays

The total phenolic content (TPC) was determined using the Folin-Ciocalteu colorimetric method [44]. Briefly, 100 μL of the resuspended extract/standard or blank was mixed with 500 μL of 1:10 (*v*/*v*) Folin–Ciocalteu reagent (Sigma-Aldrich, St. Louis, MO, USA). After 5 min, 400 μL of a solution 0.7 M of sodium carbonate (PanReac AppliChem, Barcelona, Spain) was added, and it was incubated for 2 h in a dark room at room temperature (approximately 21 °C). Then, the absorbance was measured in a plate reader (Synergy HT, BioTek Instruments, Inc.,Winoosky, GU, USA) at 760 nm against a blank of Milli-Q water. Gallic acid (Loba Chemie Pvt. Ltd., Mumbai, India) was employed to elaborate the standard curve (0.5–3 mM), and the results were expressed as milligrammes of gallic acid equivalents (GAE) per g of dry extract (DE) (mg GAE per g DE).

Total flavonoid content (TFC) was determined by the aluminium chloride colorimetric method [45]. In this assay, 80 μL of the extract was mixed with 400 μL of Milli-Q water, followed by the addition of 24 μL of a sodium nitrite solution (5%, Sigma-Aldrich, St. Louis, MO, USA). After 6 min, 48 μL of a methanolic solution of aluminium chloride hexahydrate (10%, Loba Chemie Pvt. Ltd., Mumbai, India) was added and incubated for 5 min. Next, 160 μL of a 1 M sodium hydroxide solution and 88 μL of Milli-Q water were added to the mixture, and incubated for 15 min in a dark room at room temperature. The absorbance of the final mixture was measured on a plate reader (Synergy HT, BioTek Instruments, Inc., Winoosky, GU, USA) at 510 nm against a blank of Milli-Q water. Catechin (Cayman Chemical, Ann Arbor, MICH, USA) was used to elaborate the standard curve (0.0625 mM–1.0 mM) and the results were expressed as milligrammes of catechin equivalents (CE) per g dry extract (DE) (mg CE per g DE).

The total antioxidant capacity (TAC) was determined using the ferric reducing antioxidant power (FRAP) assay [46] and the 2,2-diphenyl-1-picrylhydrazyl (DPPH) free radical method [47]. For both assays, Trolox (Sigma-Aldrich, St. Louis, MO, USA) was employed to elaborate a standard curve (50–500 µM), and the results were expressed as µmol of Trolox equivalents (TEq) per g of dry extract (µmol TEq per g DE) for both assays.

For the FRAP assay, fresh FRAP reagent was prepared daily by mixing sodium acetate trihydrate buffer (300 mM, Fisher Chemical, Waltham, MA, USA), 2,4,6-tri(2-pyridyl)-1,3,5-triazine (TPTZ) (10 mM in 40 mM HCl, Sigma-Aldrich, St. Louis, MO, USA), and FeCl_3_•6H_2_O solution (20 mM, Loba Chemie Pvt. Ltd., Mumbai, India) in ratios of 10:1:1 and incubated for 2 h at room temperature. Later, 20 μL of extract/standard or blank were mixed with 180 μL of FRAP solution, and the absorbance of the mixture was read in a plate reader at 593 nm against the blank.

For the DPPH assay, 50 μL of the resuspended extract was mixed with 400 μL of DPPH solution (0.2 mM, Alpha Aesar, Haverhill, MA, USA) in absolute methanol and 550 μL of ethanol (70%). Two blanks (B1 and B2) were prepared as follows: B1 was prepared with 400 μL of DPPH solution, 50 μL of absolute methanol, and 550 μL of ethanol (70%); while B2 was prepared with 450 μL of absolute methanol and 550 μL of ethanol (70%). The mixture was incubated for 15 min at room temperature. The absorbance of the extracts (Abs_ext_) was measured at 517 nm in a plate reader. The DPPH scavenging activity was calculated using the following equation [48]:


$$DPPH\;scavenging\;activity\;\left(\%\right)=\left(1-\frac{Abs_{ext}-B2}{B1}\right)\times 100$$

### 3.3. Bacterial Strains, Media, and Growth Conditions

The bacterial strains *Enterococcus faecium* ATCC27270, *Enterococcus faecalis* ATCC29212, *Staphylococcus aureus* ATCC25923, *Klebsiella pneumoniae* ATCC700603, *Acinetobacter baumannii* ATCC19606, *Acinetobacter baumannii* ATCC1605, *Pseudomonas aeruginosa* ATCC27853, *Enterobacter cloacae* ATCC23355, and *Escherichia coli* ATCC25922 were bought from the American Type Culture Collection (ATTC, https://www.atcc.org/). Resistant strains were donated by different research centres from Quito, Ecuador, for instance: Methicillin-resistant *Staphylococcus aureus* 333 [49,50] isolated from nasal and pharyngeal sources, and *Klebsiella pneumoniae* carbapenemase (KPC)-producing 609803 [51] were obtained from the Research Laboratories of the Universidad de Las Américas; while Vancomycin-Resistant *Enterococcus faecalis* INSPI 032 and Serine-β-lactamase (SBL) and Metallo-β-lactamase (MBL) *Escherichia coli* INSPI 033 were donated by the National Institute for Research in Public Health (INSPI, http://www.investigacionsalud.gob.ec/webs/ram/evaluacion-calidad/; accessed on 31 November 2022) to the Institute of Microbiology of the Universidad San Francisco de Quito (IM-USFQ); and finally, *Escherichia coli* extended-spectrum beta-lactamases (ESBLs) was provided from the collection of clinical isolates from the Clinical Microbiology Laboratory at the IM-USFQ [52]. All the strains used in this study, their sources, and available information are shown in Table 8. For routine cultures, bacteria were grown in Nutrient Agar (Becton, Dickinson and Company, Franklin Lakes, HI, USA), and overnight cultures were prepared in Nutrient Broth (Becton, Dickinson and Company, Franklin Lakes, HI, USA). Unless stated otherwise, all cultures were incubated at 37 °C.

### 3.4. Screening of Antibacterial Activity by the Well Diffusion Method

The antibacterial activity of 21 crude plant extracts used in the herbal infusion horchata (Table 7) was assessed by the well diffusion method as previously described [53] with the following modifications: 50 μL of the working solutions, prepared by diluting the stock solutions with Mueller Hinton Broth (MHB; Becton, Dickinson and Company, Franklin Lakes, HI, USA) to a final concentration of 50 mg/mL, were added into a circular well of 1 cm in diameter that has been bored into the surface of a Luria-Bertani (LB) agar (Becton, Dickinson and Company, Franklin Lakes, HI, USA) and sealed with 1% agarose (BioRad, Hercules, CA, USA). Working solutions were allowed complete diffusion into the LB agar for approximately 30 min. Bacterial strains were initially grown on Nutrient Agar (NA) plates and incubated for 24 h at 37 °C. Single colonies were selected to prepare an inoculum corresponding to 1.5 × 10^8^ colony-forming units (CFU)/mL using a 0.5 McFarland standard turbidity. Bacterial suspensions were diluted 1/100 and swabbed evenly onto an LB agar plate to produce a lawn of growth. Plates were incubated at 37 °C for 24 h. The inhibitory activity of the crude extracts was indicated by a zone of inhibition produced after incubation. Negative control was a solution of methanol-water (80:20, *v*/*v*) used to discard any influence of methanol in the inhibitory activity of the crude extracts. To test sterility, working solutions were inoculated in NA and incubated at 37 °C for 24 h.

### 3.5. Antibacterial Activity by Microdilution Assay

Minimum inhibitory concentration (MIC) and minimal bactericidal concentration (MBC) from 12 crude plant extracts that showed antibacterial activity in the initial screening were then tested using the broth microdilution technique according to the Clinical and Laboratory Standards Institute [54] recommendations with some modifications. Single colonies, from a 24-h culture, were selected to prepare an inoculum corresponding to 1.5 × 10^8^ CFU/mL using a 0.5 McFarland standard turbidity. The suspensions were adjusted to a final micro-organism density of 5 × 10^5^ CFU/mL. Working solutions of crude plant extracts were prepared by diluting the stock solutions with MHB to a final concentration of 4 mg/mL. A 96-well U-shaped microplate (Tecan Group Ltd., Mannedorf, Switzerland) was filled in the first wells with 100 µL of working solution and allowed methanol evaporation for 30 min. Then, 100 µL of MHB was added to the first well mix and 100 µL was taken from the first well to do 2-fold serial dilutions. After that, 100 µL of bacterial suspension previously prepared was added to each well. The microplate was incubated at 37 °C between 16 and 20 h. The final concentrations of plant crude extracts were 31.2–1000 µg/mL. Negative control was a solution with a final concentration of methanol-water (80:20, *v*/*v*) between 0.5 and 0.9% used to discard any influence of methanol in the inhibitory activity of the crude extracts. This control was treated similarly to the samples. A well with MHB plus inoculum was used as a growth control (or positive control), and a medium with no inoculum was applied to control sterility. Ciprofloxacin (Sigma-Aldrich, St. Louis, MO, USA) was used as a positive control, with concentrations ranging from 0.09 to 3 µg/mL. The MIC was measured by the unaided eye by comparing the results obtained with the negative control. The MIC is defined as the lowest concentration of a drug (extract) that inhibits the visible growth of an organism after overnight incubation [19]. The minimum bactericidal concentration (MBC) was determined following the MIC assay by plotting 3 µL of samples from clear wells onto NA plates without extract. The agar plates were incubated at 37 °C for 24 h. MBC was estimated as the least sample concentration, where no visible growth was observed. MBC is defined as the minimum bacterial concentration required to completely kill the original inoculums [19]. All MIC and MBC assays were performed in triplicate in at least two independent experiments.

### 3.6. Biofilm Inhibition Assay

The effect of crude plant extracts against susceptible and resistant bacterial strains was further evaluated against biofilm formation. An overnight culture of the bacterial strains was used to prepare a bacterial suspension with a final concentration of 5 × 10^5^ CFU/mL in MHB. Then, 100 µL of the bacterial suspension was added to 96-well flat bottom plates (Tecan Group Ltd., Mannedorf, Switzerland) and supplemented with crude plant extracts at 1× and 2× MIC concentrations [55]. Plates were then incubated for 24 h at 37 °C under aerobic conditions. Subsequently, the media was removed from the wells, and each well was washed three times with Dulbecco’s phosphate buffered saline (PBS, pH 7.4; Sigma-Aldrich, St. Louis, MO, USA), and each biofilm sample in the well was fixed with 200 µL of methanol (99% *v*/*v*; Sigma-Aldrich, St. Louis, MO, USA) for 15 min. Crystal violet staining (0.1% *w*/*v*; Sigma-Aldrich, St. Louis, MO, USA) was performed for 10 min [55,56], followed by four washing steps with distilled water. Ethanol (95% *v*/*v*; Sigma-Aldrich, St. Louis, MO, USA) was used to dissolve the biofilm, and its inhibition was measured by spectrophotometry in the ELISA Elx808 spectrophotometer (BioTek, Winooski, GU, USA) at an optical density of 570 nm [55] by comparing with positive controls (bacteria growth in media only). Finally, the percentage of biofilm inhibition was calculated. The assays were performed in triplicate in three independent experiments. As previously mentioned, a negative control was a solution of methanol-water (80:20, *v*/*v*), and a well with medium without inoculum was applied as a control for sterility.

### 3.7. Biofilm Eradication Assay

From an overnight culture of the bacterial strains, 30 µL (1 × 10^5^ CFU/mL) was taken and added to a 96-well flat bottom plate, followed by the addition of 150 µL of MHB [57]. Then, the plate was incubated for 48 h at 37 °C under aerobic conditions for biofilm formation. Subsequently, the MHB was removed from the wells, and a wash with PBS (pH 7.4) was performed. The biofilms were then supplemented with crude plant extracts at 1× and 2× MIC values in flesh media [55]. Plates were again incubated for 24 h at 37 °C under aerobic conditions. Three washes were performed with PBS (pH 7.4), and crystal violet staining (0.1% *w*/*v*) was performed for 10 min, followed by four washes with distilled water. Ethanol (95% *v*/*v*) was then used to dissolve the biofilm, and its eradication was measured by spectrophotometry at an optical density of 570 nm, by comparing it with positive controls (bacteria growth in media only). Finally, the percentage of biofilm eradication was calculated [55,56,58]. The assays were performed in triplicate in three independent experiments, using the same negative, sterile, and positive controls previously described.

### 3.8. HPLC-DAD-MS/MS Analysis for Polyphenolic Composition

Dry hydroalcoholic extracts were prepared by resuspending 20 mg of dry extract in 1 mL of methanol/water solution (80:20, *v*/*v*) for analysis by high-performance liquid chromatography (HPLC). The HPLC system consisted of a Vanquish (Thermo Fisher Scientific, Massachusetts, MA, USA) fitted with a dual pump and diode-array detection (DAD) coupled with an LTQ-XL (Thermo Fisher Scientific, Massachusetts, MA, USA) controlled by Xcalibur Software. The separation was achieved in an Accucore Vanquish C18 column (1.5 μm, 100 × 2.1 mm; Merck KGaA, Darmstadt, Germany) thermostat at 35 °C. The mobile phase consisted of a solution of 0.1% formic acid (A) and acetonitrile (B). The elution gradient established was of the isocratic type 2% B, 0–4 min; 4% B, 4–22 min; 40% B, 22–32 min; 70% B, 32–40 min; 2% B, 40–45 min, and rebalanced the column to the initial conditions of the solvent. The flow rate was 0.2 mL/min, and the injection volume was 50 µL. Double-line detection was carried out in DAD at 220, 280, 330, and 370 nm as preferred wavelengths, and the mass spectrometer (MS) operated in positive and negative ionisation modes. Spectra between 50 m/z and 2000 m/z were recorded. The ESI (electrospray ionisation) conditions in negative ionisation mode were: capillary temperature of 275 °C, source voltage and capillary voltage of 5 kV and −21 V, respectively, tube lens −67, and the positive ionisation mode were: capillary temperature of 275 °C, source voltage of 4.5 kV, capillary voltage of 18 V, and tube lens −70. The individual compounds were tentatively identified from their mass and ultraviolet (UV) spectra and compared with data reported in the literature and the MzCloud database (https://www.mzcloud.org/; accessed on 20 September 2022).

### 3.9. Statistical Analyses

All data of the present study were performed in triplicate in at least two independent experiments. Due to the non-normal distribution of the data set, a nonparametric test was applied; more specifically, the Wilcoxon nonparametric test was used for pairwise comparison between control and treated samples in biofilm inhibition and eradication assays. All data analyses were realised in R Studio version 4.0 (https://www.rstudio.com/products/rstudio/download/; accessed on 15 October 2022) using several R packages (“ggpubr”, “rstatixs”, “openxlsx”, and the “tidyverse” set of packages) [59,60]. Finally, all *p*-values < 0.05 were considered significant.

## 4. Discussion

Plants are an important source of new chemical scaffolds, which serve as templates for the derivation of novel antibiotics. In this study, we evaluated the antibacterial activity of 21 crude methanol extracts used in the horchata drink, over the growth of bacterial pathogens of clinical importance, and related their antibacterial activity with the extract’s chemical composition. Our initial screening identified inhibitory activity against both gram-positive and gram-negative bacteria (Table 2); however, only the ones with a minimum inhibitory concentration (MIC), equal to or lower than 1000 µg/mL, were considered effective and were further selected (Table 4). The MIC is considered the gold standard to determine the susceptibility of organisms to antimicrobials [19], and it is advised that only MIC and MBC values should be used to compare results between different studies [61]. In this study, *Cinnamomum* sp. and *P. odoratissimum* exhibited effective MIC values only against gram-positive strains (Table 4).

*Cinnamomum* sp., a genus within the botanical family *Lauraceae*, is one of the common natural products used in medical applications due to its antioxidative, anti-inflammatory, cardioprotective, anticancer, antidiabetic, and antimicrobial properties [62]. In our study, *Cinnamomum* sp. extract showed better activity against gram-positive bacteria (Table 4), in agreement with another study using methanol extracts of the bark of the plant [63]. Other studies have reported different species of *Cinnamomum* extracts showing inhibitory activity against a wide range of bacteria [64,65]; however, the methods of extractions, the MIC effective values and parts of the plants used differ from the ones used in our study, as reviewed by Bhatia et al. [9]. Importantly, our results indicate a MIC value of *Cinnamomum* sp. towards MRSA 333 (250 µg/mL) clinical strains, two times lower than the value reported by Buru et al. [63] (MIC 650 µg/mL) when testing methanol/dimethyl sulfoxide (DMSO) extracts of the stem bark of different *Cinnamomum* species. In contrast, Manandhar et al. [66] reported a MIC value of 12.25 mg/mL on *S. aureus* ATCC 25923 and no activity towards the MRSA strain when assessing the methanol/DMSO extracts of the bark part of *C. tamala*. Overall, the MIC values for *S. aureus* and MRSA 333 reported in this study are better than the ones reported in the literature when working with *Cinnamomum* methanolic extracts.

The *Pelargonium* genus belongs to the family *Geraniaceae* and it is widely known for its health benefits. Studies using *Pelargonium* sp. essential oils described their anti-inflammatory, antioxidant, and antimicrobial properties [67,68,69,70,71]. The antimicrobial activity of *P. odoratissimum* volatile oils [67,70,72,73] and aqueous extract [71] have been reported to show inhibitory activity over the growth of bacteria and fungi. Overall, these studies reported *P. odoratissimum* volatile oils and aqueous extract with activity towards *S. aureus*; however, the lack of data about MIC values hinders comparisons between these studies and our work. It is noteworthy that there is no information about the antimicrobial activity of *P. odoratissimum* using methanolic extracts, this being the first report of this nature. In our study, *P. odoratissimum* showed inhibitory activity *against S. aureus* (MIC 500 µg/mL) yet no effective MIC value against the MRSA 333 strain (>1000 µg/mL) was observed. This may be due to a strain-dependent inhibitory activity, an effect that was reported by others when observing different inhibitory activities of the same phenolic compounds towards *E. coli* and *E. coli* O157:H7 [74]. Overall, the little inhibitory activity observed in the gram-negative bacteria (Table 2) may be attributed to the presence of an outer membrane of lipopolysaccharides that surround the cell wall of gram-negative bacteria. The absence of this membrane in gram-positive bacteria may contribute to a greater permeability of bioactive phytochemicals [75].

The chemical analysis identified in *Cinnamomum* sp. and *P. odoratissimum* molecules, such as phenolic acids, several flavonoids, and tannins, that could be perfectly responsible for the antibacterial activity detected [2]. However, both plants showed a very different content of flavonoids. *Cinnamomum* sp. not only had a higher number of flavonoids (compared to *P. odoratissimum*) but also a much higher fraction (TFC/TPC). It is important to note that the majority of chemical composition studies carried out in *Cinnamomum* sp. and *P. odoratissimum* have been performed using essential oils. Articles exploring the chemical composition or metabolome in hydroalcoholic extracts are a minority, especially for *Pelargonium* sp. In the case of *P. odoratissimum*, no article was found studying the chemical composition of hydroalcoholic extracts (only for *Pelargonium sidoides* [76,77] and *Pelargonium graveolens* [78]). Likewise, studies about the antioxidant capacity of *P. odoratissimum* were not found. In this sense, our work is a primary contribution. A few more articles were found studying the chemical composition of hydroalcoholic extracts of different *Cinnamomum* species using LC-MS/MS [24,28,79,80]. Moreover, the high antioxidant capacity of *Cinnamomum* sp. (DPPH and FRAP > 1000 µmol TEq per g DE) (Table 1) reported in this study is in agreement with previous reports [81,82]. This activity is associated with elevated levels of phenolic compounds, especially tannins, which are the strongest radical scavengers compared to other phenolic compounds [81].

In *Cinnamomum* sp. besides epicatechin tannins, we also found several cinnamaldehyde derivatives, which among other secondary metabolites are common in this genus (Table 5) [24]. Interestingly, we identified prehelminthosporol, a derivate of the Sativene sesquiterpenoids which have been obtained mainly from the endophytic fungi *Cochliobolus*, *Drechslera*, and *Bipolaris* sp. [35,83]. Endophytic fungi live inside plant tissues either for a short period of time or throughout their life, without causing visible damage [84]. Prehelminthosporol is a cytotoxin with known antimicrobial and antiplasmodial properties [83]. The endophytic fungus from the genus *Drechslera* has been found in *Cinnamomum camphora* [85]. To the authors’ best knowledge, this is the first time that this molecule is reported as part of the chemical composition of *Cinnamomum* sp. Thus, we believed that the presence of prehelminthosporol in the *Cinnamomum* methanol extract may be either due to the presence of an endophytic fungi in the bark part of the plant or is perhaps the result of a cross-contamination from other plants that were in contact with the *Cinnamomum* plant when it was bought from commercial places in Ecuador.

The mechanism of action proposed for cinnamaldehyde is related to cell destruction [86,87], while for tannins, it is suggested they act by inhibiting or inactivating bacterial virulence factors (e.g., proteins, enzymes, microbial adhesins, and toxins) [2,88], resulting in the inhibition of a bacterial critical process, such as bacterial metabolism [89]. Our results showed antibacterial activity for both *S. aureus* and MRSA 333 (Table 4), suggesting that the mechanism of action of *Cinnamomum* sp. might be related to cell destruction and/or the inactivation of bacterial virulence factors, regardless of showing high affinity or not to the non-native penicillin-binding protein (PBP2a) responsible for *S. aureus* methicillin-resistance [90], as others have proposed [63]. It is noteworthy that the antibacterial activity found in the *Cinnamomum* extract may also be influenced by the presence of the cytotoxic prehelminthosporol molecule.

The chemical composition of *P. odoratissimum* is very similar, at least in the majority of compounds reported previously in this genus [91]. The crude extract of this plant is enriched in gallocatechin and epigallocatechin tannins, some flavonoids, and gallic acid and derivatives (Table 6). Moreover, *P. adoratissimum* has minor amounts of luteolin, quercetin, and their derivatives. Importantly, in both plant extracts, we identified tannins. However, in *P. odoratissimum*, the tannins identified were gallocatechin derivatives while in *Cinnamomum* sp., they were epicatechin derivatives. In addition, several gallic acid derivatives were found in *P. odoratissimum*, in contrast to *Cinnamomum* sp., where no gallic acid derivatives were found.

In a recent study by Neumann et al. [92], when exploring the antimicrobial activity of several plant extracts against multidrug-resistant *E. coli*, they found that the inhibitory activity was mostly related to the tannin concentration rather than flavonoids (especially in alcoholic extracts). This finding is consistent with the presence of tannins in *both Cinnamomum* sp. and *P. odoratissimum* plants (Table 5 and Table 6). Unfortunately, we did not quantify the total tannin amount separately from the total polyphenol content. In addition, we found that the two plant extracts exert bactericidal activity over the growth of gram-positive bacteria (Table 4, see MBC). It has been reported that flavonoids do not exhibit bactericidal activity, but rather the formation of aggregates (bacteriostatic activity) is responsible for CFU reduction counts in *S. aureus* strains [93,94,95,96], suggesting that the bactericidal activity observed by *Cinnamomum* sp. and *P. odoratissimum* may be due to the presence of tannins and other molecules (such as cinnamaldehyde and derivatives) instead of flavonoids. If the antibacterial effects were mainly related to flavonoids, then *Melissa officinalis* or even *Ocimum basilicum* instead of *P. odotosissimum* should be found with better antimicrobial activity. On the other hand, it has been reported that the inhibitory effect of tannins in gram-negative bacteria is lower than in gram-positive bacteria [89], which may explain the results reported in this study (Table 2). Quantifying secondary metabolites over the total phenolic content will be the focus of future work.

In this study, the two plants with the best antibacterial effects (*Cinnamomum* sp. and *P. odoratissimum*) (Table 4) showed a higher concentration of polyphenols (TPC) and higher antioxidant capacities (measured by FRAP and DPPH) (Table 1), in agreement with other studies [7,97]. However, even though *Melissa officinalis* showed high levels of TPC, FRAP, and DPPH (Table 1), no antibacterial activity by this plant extract was observed (Table 1 and Table 2). In fact, when comparing the amount of total phenolic content (TPC) (Table 1) with the inhibitory activity of the crude extract (Table 2), there was not a direct association between the amount of TPC and the presence of inhibitory activity. In other words, not necessarily the plants with low TPC, nor even those with high TPC, FRAP, and DPPH, have the best or non-inhibitory activity (Table 2). It has been noticed that the antimicrobial activity of a crude extract is not always attributed solely to a single bioactive compound, but also to its combination with other secondary metabolites [2]. In addition, a single bioactive compound might be working in a complementary, synergistic, and/or additive manner with multiple phytochemicals. Thus, it may be that the crude extracts with high TPC content and no antimicrobial activity required other phytochemicals to work synergistically and that the inhibitory activity of samples with low TPC content is due to the presence of other bioactive compounds.

Overall, it was noticed that most studies with reports of antimicrobial activity exerted by *Cinnamomum* sp. and *P. odoratissimum* did not specify or characterise the phytochemicals contained in the extracts, while in our study we complemented the antibacterial activity found with a chemical characterization of the majoritarian compounds with suggestions of possible mechanisms of action. We note that other studies [9,16,98] have reported antimicrobial activity by some of the plants tested in this study for, which we did not observe inhibitory effects on bacterial growth (Table 2). The main difference bewteen studies is the solvent used. In our work, we have evaluated the antimicrobial activity of methanol-derived extracts in contrast with others using acetone, hexane, water, and DMSO as solvents. The solvent will influence the type and concentration of the bioactive compounds found, which relates directly to the antimicrobial activity. For instance, the extract of *Ocimum tenuiflorum*, reported by Srichok et al. [16], with antibacterial activity towards *S. aureus*, used DMSO as a solvent. In addition, other factors that could be influencing the presence of activity are differences in chemical compositions and the number of phytochemicals in plant species of the same genus but obtained from different geographic areas.

*Cinnamomum* sp. and *P. odoratissimum* extracts differed in their capacity to inhibit biofilm formation against *S. aureus*, which was already expected and reported in previous studies with *Cinnamomum* sp. essential oil and ethanol extracts [65,99]. To the best of the authors’ knowledge, there are no studies that reported antibiofilm activities by *P. odoratissimum* extracts in bacteria, although some authors reported anti-*Candida* activity through *Pelargonium graveolens* oil-free [100]. As recently reviewed by Didehdar et al. [101], the potent antibacterial properties of *Cinnamomum* and its derivatives (such as cinnamaldehyde and tannins) could be promising as agents for inhibiting microbial biofilm-associated infections. Previous studies demonstrated that cinnamaldehyde concentrations higher than 1 mg/mL were able to eliminate preformed *S. aureus* biofilms [102]. Kot et al. [103] reported an antibiofilm effect on preformed MRSA biofilms after 48 h of treatment by cinnamaldehyde, showing eradication values between 53 and 82%. Finally, in another study, Rubini et al. [104] showed a disruption of 60–80% in MRSA biofilms by *Cinnamomum* sp. essential oil. According to these studies, *Cinnamomum* essential oils or extracts and their derivatives were able to prevent *S. aureus* initial adhesion and biofilm formation through the downregulation of various MRSA genes [101,102,103]. However, the biofilm inhibition range of these plant extracts was lower than the values reported in the previous studies. It is important to mention that these authors used petroleum ether and ethanol as solvents for the plant extracts, and no evaluation of solvent controls was reported, which could contribute to the high biofilm inhibition values obtained [105].

Meanwhile, Ben Abdallah et al. [106] evaluated biofilm eradication, reporting high values ranging from 18.3 to 98.0%. However, these plant essential oils were evaluated by the authors in their pure state (100% pure), as previously described by Jadhav et al. [107] and potentially justifying the higher eradication values reported. In the present study, only 2 g of the ground sample from each plant was used in the extraction, with 20 mL of a methanol/water solution (80:20, *v*/*v*), being so strongly diluted when applied against biofilms. The high cell density, the presence of persister cells, and the amount of extracellular polymeric substances (EPS) in the *S. aureus* biofilms could explain the lack of significant biofilm eradication results and also the low biofilm inhibition values [108,109]. As postulated by Costa et al. [110], the bioactive compounds need to be identified and purified, being further evaluated against *S. aureus* biofilms at higher concentrations. These limitations must be solved in future studies of biofilm assays.

## 5. Conclusions

In conclusion, after evaluating 21 plants commonly used in the preparation of horchata, we have identified *Cinnamomum* sp. and *P. odoratissimum* as responsible for the antimicrobial activity attributed to this medicinal drink. *Cinnamomum* sp. and *P. odoratissimum* methanolic extracts showed the best antibacterial activity, with *Cinnamomum* inhibiting the growth of *S. aureus* (MIC 250 µg/mL) and MRSA 333 clinical strain (MIC 250 µg/mL), and *P. odoratissimum* only inhibiting the growth of *S. aureus* (MIC 500 µg/mL). The chemical characterisation of *Cinnamomum* sp. and *P. odoratissimum* indicated the presence of phenolic acids, as well as several flavonoids, and tannins. Specifically, in *Cinnamomum* sp., the main molecules identified were epicatechin tannins, cinnamaldehyde, and prehelminthosporol while in *P. odoratissimum*, the molecules identified were gallocatechin and epigallocatechin tannins, some flavonoids, and gallic acid and derivatives. Moreover, *Cinnamomum* sp. extract exhibited partial biofilm inhibition against *S. aureus* and MRSA 333, whereas *P. odoratissimum* extract only demonstrated inhibition towards *S. aureus*. Overall, these findings revealed which of the plants used in horchata are responsible for the antibacterial activity attributed to this herbal drink and may provide further validation of *Cinnamomum* sp. and *P. odoratissimum* secondary metabolites as scaffolds to be explored in drug development to treat infectious diseases caused by *S. aureus* and MRSA, considered one of the major pathogens causing skin and soft tissue infections.

## Figures and Tables

**Table 1:** Total phenolic content, total flavonoid content, and total antioxidant capacity of 21 crude plant extracts used in the herbal infusion horchata.

N	Scientific Name	TPC (mg GAE per g DE)	TFC (mg CE per g DE)	TAC (μmol TEq per g DE)	
				FRAP	DPPH
1	*Amaranthus hybridus*	28.7 ± 0.9	10.9 ± 1.0	87.1 ± 5.5	136.3 ± 5.0
2	*Ocimum tenuiflorum*	36.2 ± 1.8	14.9 ± 0.3	155.6 ± 6.5	177.8 ± 10.5
3	*Ocimum basilicum* L.	91.3 ± 2.9	87.4 ± 2.0	705.0 ± 32.2	697.8 ± 11.9
4	*Borago officinalis* L.	48.8 ± 2.6	31.9 ± 1.5	238.7 ± 9.0	263.6 ± 25.9
5	*Aloysia triphylla* (L’Hér.) Britton	98.3 ± 1.9	77.6 ± 3.4	622.9 ± 7.3	761.3 ± 33.1
6	*Adiantum concinnum* Humb. and Bonpl. ex Wild.	105.2 ± 3.0	78.8 ± 2.1	438.8 ± 18.9	1036.3 ± 91.2
7	*Equisetum bogotense* Kunth	224.4 ± 6.3	19.9 ± 0.6	171.6 ± 4.1	228.9 ± 19.5
8	*Cinnamomum* sp.	382.1 ± 28.1	180.5 ± 19.9	1471.3 ± 18.1	2894.3 ± 272.6
9	*Aerva sanguinolenta* L. (Blume).	50.3 ± 1.8	23.0 ± 1.3	192.9 ± 2.2	284.0 ± 21.7
10	*Stevia rebaudiana* (Bertoni) Bertoni	149.5 ± 9.7	98.6 ± 2.9	601.3 ± 26.6	621.8 ± 79.6
11	*Cymbopogon citratus* (DC.) Stapf	31.6 ± 3.4	14.1 ± 0.5	117.2 ± 4.7	164.3 ± 10.5
12	*Citrus x aurantium* L.	71.3 ± 0.9	34.7 ± 0.7	256.6 ± 2.2	341.0 ± 25.1
13	*Plantago major* L.	35.0 ± 1.5	17.6 ± 0.2	123.2 ± 6.1	183.5 ± 7.6
14	*Pelargonium odoratissimum* (L.) L’Hér.	258.2 ± 14.4	31.0 ± 1.4	901.8 ± 38.7	2777.9 ± 17.1
15	*Althaea officinalis* L.	33.8 ± 1.1	5.9 ± 0.2	71.0 ± 4.9	79.9 ± 6.6
16	*Matricaria chamomilla* L.	32.5 ± 1.7	9.9 ± 0.2	107.4 ± 4.5	154.9 ± 1.1
17	*Mentha x piperita* L.	52.2 ± 0.9	45.4 ± 1.0	198.5 ± 6.7	245.7 ± 23.7
18	*Origanum vulgare* L.	96.1 ± 4.1	56.1 ± 2.8	507.0 ± 39.7	768.5 ± 12.9
19	*Fuchsia loxensis* Kunth	67.5 ± 1.0	29.8 ± 1.1	317.6 ± 8.0	533.4 ± 44.9
20	*Melissa officinalis* L.	299.6 ± 10.3	256.4 ± 3.3	1582.4 ± 67.0	1965.2 ± 84.3
21	*Viola odorata* L.	74.0 ± 1.2	31.9 ± 1.0	462.9 ± 34.8	568.1 ± 47.7

Notes: Results are expressed as mean ± standard deviation (SD). TPC: Total phenolic content, TFC: Total flavonoid content, TAC: total antioxidant capacity. FRAP: Ferric reducing/antioxidant power test, DPPH: 2,2-diphenyl-1(2,4,6-trinitrophenyl) hydrazyl free radical method.

**Table 2:** Antibacterial activity of 21 crude plant extracts used in the herbal infusion horchata against susceptible bacterial strains of clinical importance determined by the well diffusion method.

N	Plant Scientific Name	Susceptible Bacterial Strains (Inhibition Zone Diameter around Circular Well in mm)							
		*E. faecium*	*E. faecalis*	*S. aureus*	*K. pneumoniae*	*A. baumannii*	*P. aeruginosa*	*E. cloacae*	*E. coli*
1	*Amaranthus hybridus*	-	-	-	-	-	-	-	-
2	*Ocimum tenuiflorum*	-	-	-	-	-	-	-	-
3	*Ocimum basilicum* L.	-	10	6	-	-	-	-	-
4	*Borago officinalis* L.	-	6	2	-	-	-	-	-
5	*Aloysia triphylla* (L’Hér.) Britton	-	-	2	-	4	-	-	-
6	*Adiantum concinnum* Humb. and Bonpl. ex Wild	-	6	10	-	7	-	-	-
7	*Equisetum bogotense* Kunth	-	-	-	-	-	-	-	-
8	*Cinnamomum* sp.	-	-	17	-	5	-	5	-
9	*Aerva sanguinolenta* L. (Blume)	-	-	-	-	-	-	-	-
10	*Stevia rebaudiana* (Bertoni) Bertoni	-	-	7	-	-	-	-	-
11	*Cymbopogon citratus* (DC.) Stapf	-	-	-	-	-	-	-	6
12	*Citrus x aurantium* L.	-	-	-	-	-	-	-	-
13	*Plantago major* L.	-	-	8	-	-	-	-	-
14	*Pelargonium odoratissimum* (L.) L’Hér.	-	-	15	3	-	-	-	10
15	*Althaea officinalis* L.	-	-	-	-	-	-	-	-
16	*Matricaria chamomilla* L.	-	-	7	-	-	-	-	-
17	*Mentha x piperita* L.	-	6	9	-	-	-	-	-
18	*Origanum vulgare* L.	-	9	11	-	-	-	-	-
19	*Fuchsia loxensis* Kunth	-	-	-	-	-	-	-	-
20	*Melissa officinalis* L.	-	-	-	-	-	-	-	-
21	*Viola odorata* L.	-	-	-	-	-	-	-	-
	Negative control	-	-	-	-	-	-	-	-

Notes: Negative control is methanol-water (80:20, *v*/*v*). (-) no inhibitory activity was observed.

**Table 3:** Antibacterial activity of crude plant extracts used in the herbal infusion horchata against resistant bacterial strains of clinical importance determined by the well diffusion method.

N	PlantScientific Name	Resistant Bacterial Strains (Inhibition Zone Diameter around Circular Well in mm)					
		MRSA 333	*E. faecalis* INSPI 032	*K. pneumoniae* KPC 609803	*E. coli* ESBL	*E. coli* INSPI 033	*A. baumannii* ATCC1605
1	*Ocimum basilicum* L.	-	-	NT	NT	NT	NT
2	*Borago officinalis* L.	-	-	NT	NT	NT	NT
3	*Aloysia triphylla* (L’Hér.) Britton	-	NT	NT	NT	NT	-
4	*Adiantum concinnum* Humb. and Bonpl. ex Wild	-	-	NT	NT	NT	-
5	*Cinnamomum* sp.	14	NT	NT	NT	NT	-
6	*Stevia rebaudiana* (Bertoni) Bertoni	7	NT	NT	NT	NT	-
7	*Cymbopogon citratus* (DC.) Stapf	NT	NT	NT	-	-	NT
8	*Plantago major* L.	-	NT	NT	NT	NT	NT
9	*Pelargonium odoratissimum* (L.) L’Hér.	4	NT	-	-	-	NT
10	*Matricaria chamomilla* L.	-	NT	NT	NT	NT	NT
11	*Mentha x piperita* L.	-	-	NT	NT	NT	NT
12	*Origanum vulgare* L.	-	-	NT	NT	NT	NT
	Negative control	-	-	-	-	-	-

Notes: Negative control is methanol-water (80:20, *v*/*v*). (-) no inhibitory activity was observed. “NT”: not tested as no inhibitory activity was observed with its respective susceptible strain.

**Table 4:** Minimum inhibitory concentrations (MICs) and minimal bactericidal concentrations (MBCs) of crude plant extracts used in the herbal infusion horchata against bacterial strains of clinical importance.

N	Plant Scientific Name	Bacterial Strains MIC; MBC (Values Are in µg/mL)							
		*E. faecalis*	*S. aureus*	*K. pneumoniae*	*A. baumannii*	*E. cloacae*	*E. coli*	MRSA 333	*E. faecalis* INSPI 032
1	*Ocimum basilicum* L.	>1000	>1000	NT	NT	NT	NT	NT	NT
2	*Borago officinalis* L.	>1000	>1000	NT	NT	NT	NT	NT	NT
3	*Aloysia triphylla* (L’Hér.) Britton	NT	>1000	NT	>1000	NT	NT	NT	NT
4	*Adiantum concinnum* Humb. and Bonpl. ex Wild	>1000	>1000	NT	>1000	NT	NT	NT	NT
5	*Cinnamomum* sp.	NT	**250; 250**	NT	>1000	>1000	NT	**250; 500**	NT
6	*Stevia rebaudiana* (Bertoni) Bertoni	NT	>1000	NT	NT	NT	NT	>1000	NT
7	*Cymbopogon citratus* (DC.) Stapf	NT	NT	NT	NT	NT	>1000	NT	NT
8	*Plantago major* L.	NT	>1000	NT	NT	NT	NT	NT	NT
9	*Pelargonium odoratissimum* (L.) L’Hér.	NT	**500; 1000**	>1000	NT	NT	>1000	>1000	NT
10	*Matricaria chamomilla* L.	NT	>1000	NT	NT	NT	NT	NT	NT
11	*Mentha x piperita* L.	>1000	>1000	NT	NT	NT	NT	NT	NT
12	*Origanum vulgare* L.	>1000	>1000	NT	NT	NT	NT	NT	NT
-	Ciprofloxacin	1.5	0.38	0.15	0.75	<0.09	<0.09	NT	NT

Notes: MBCs were determined for MIC values ≤ 1000 µg/mL. “NT”: not tested as no inhibitory activity was observed in the well diffusion assay. Ciprofloxacin was used as a positive control for all bacterial strains.

**Table 7:** List of horchata plants and parts of the plants used in this study.

N	Common Name	Scientific Name	Part of the Plant
1	Ataco	*Amaranthus hybridus*	Flower
2	Albahaca negra o dulce	*Ocimum tenuiflorum*	Leaf, stalk
3	Albahaca blanca o salada	*Ocimum basilicum* L.	Leaf, stalk
4	Borraja	*Borago officinalis* L.	Plant without root
5	Cedrón	*Aloysia triphylla* (L’Hér.) Britton	Leaf
6	Culantrillo	*Adiantum concinnum* Humb. and Bonpl. ex Wild.	Leaf
7	Cola de caballo	*Equisetum bogotense* Kunth	Branch
8	Canela	*Cinnamomum* sp.	Bark
9	Escancel	*Aerva sanguinolenta* L. (Blume).	Plant without root
10	Stevia	*Stevia rebaudiana* (Bertoni) Bertoni	Leaf
11	Hierba luisa	*Cymbopogon citratus* (DC.) Stapf	Leaf
12	Hoja de naranja	*Citrus x aurantium* L.	Leaf
13	Llantén	*Plantago major* L.	Plant without root
14	Malva olorosa	*Pelargonium odoratissimum* (L.) L’Hér.	Leaf
15	Malva blanca	*Althaea officinalis* L.	Leaf
16	Manzanilla	*Matricaria chamomilla* L.	Plant without root
17	Menta	*Mentha x piperita* L.	Leaf, stalk
18	Orégano dulce	*Origanum vulgare* L.	Leaf
19	Pena pena	*Fuchsia loxensis* Kunth	Plant without root
20	Toronjil	*Melissa officinalis* L.	Leaf, stalk
21	Violeta	*Viola odorata* L.	Flower, leaf

**Table 8:** List of all micro-organisms used in this study, their sources, and available information.

N	Microorganisms	Source	Available Information	Reference
1	*Enterococcus faecium* ATCC27270	*Enterococcus faecium* (Orla-Jensen) Schleifer and Kilpper-Balz (ATCC27270)	Isolate contains enterococcal bacteriocins, and it is a whole-genome sequenced clinical isolate.	ATCC
2	*Enterococcus faecalis* ATCC29212	*Enterococcus faecalis* (Andrewes and Horder) Schleifer and Kilpper-Balz (ATCC29212)	The isolate was obtained from a human urine sample, and it is a whole-genome sequenced clinical isolate.	ATCC
3	*Staphylococcus aureus* ATCC25923	*Staphylococcus aureus* subsp. *aureus* Rosenbach (ATCC25923)	The strain was obtained in Seattle in 1945, and it is a whole-genome sequenced clinical isolate.	ATCC
4	*Klebsiella pneumoniae* ATCC700603	*Klebsiella quasipneumoniae* Brisse et al. (ATCC700603)	It is a whole-genome sequenced bacterium that was isolated from the urine of a hospitalised patient in Richmond, Virginia. This bacterium produces beta-lactamase SHV-18 and can be used as a CLSI quality control strain for antimicrobial susceptibility testing.	ATCC
5	*Acinetobacter baumannii* ATCC19606	*Acinetobacter baumannii* Bouvet and Grimont (ATCC19606)	The isolate was obtained from a human urine sample, and it is a whole-genome sequenced clinical isolate that can be used as a quality control strain for antimicrobial susceptibility testing.	ATCC
6	*Acinetobacter baumannii* ATCC1605	*Acinetobacter baumannii* (ATCCBAA-1605)	The isolate was obtained from a human sputum of military personnel returning from Afghanistan entering a Canadian hospital and can be used for antimicrobial resistance research or drug development.	ATCC
7	*Pseudomonas aeruginosa* ATCC27853	*Pseudomonas aeruginosa* (Schroeter) Migula (ATCC27853)	The isolate was obtained from a hospital blood specimen in 1971, and this whole-genome sequenced bacterial strain has applications in susceptibility testing.	ATCC
8	*Enterobacter cloacae* ATCC23355	*Enterobacter cloacae* subsp. *cloacae* (Jordan) Hormaeche and Edwards, subsp. nov. (ATCC23355)	Isolate produces cephalosporinase beta-lactamase II, and this whole-genome sequenced bacterial strain is a well-known bacteriophage host.	ATCC
9	*Escherichia coli* ATCC25922	*Escherichia coli* (Migula) Castellani and Chalmers (ATCC25922)	*Escherichia coli* human isolate strain Seattle 1946 is a whole-genome sequenced quality control strain that does not produce verotoxin and can be used as a CLSI control strain for antimicrobial susceptibility testing.	ATCC
10	Methicillin-resistant *Staphylococcus aureus* 333	Universidad de Las Américas (UDLA)	Isolated strains obtained from nasal and pharyngeal sources in Ecuadorian patients.	[49,50]
11	*Klebsiella pneumoniae* carbapenemase (KPC)-producing 609803	UDLA	The isolate was obtained from human samples at the Research Laboratories of the Universidad de Las Américas (UDLA).	[51]
12	Vancomycin-Resistant *Enterococcus faecalis* INSPI 032	Instituto Nacional de Investigación en Salud Pública (INSPI)	The isolate was obtained from human samples, and it was donated by INSPI.	INSPI
13	Metallo-β-lactamase (MBL) *Escherichia coli* INSPI 033	INSPI	The isolate was obtained from human sample, and it was donated by the National Institute for Research in Public Health (INSPI) in Ecuador.	INSPI
14	*Escherichia coli* extended-spectrum beta-lactamases (ESBLs)	Institute of Microbiology at Universidad San Francisco de Quito (IM-USFQ)	The isolate was obtained from human faecal sample at the Clinical Microbiology Laboratory of the IM-USFQ.	[52]

## Data Availability

Not applicable.

## Supplementary Materials

The following supporting information can be downloaded at: https://www.mdpi.com/article/10.3390/molecules28020693/s1, Table S1: Biofilm inhibition of bacterial strains of clinical importance by plant extracts used in the herbal infusion horchata; Table S2: Biofilm eradication of bacterial strains of clinical importance by plant extracts used in the herbal infusion horchata.
