# Supplementary material for: Cinnamomum sp. and Pelargonium odoratissimum as the Main Contributors to the Antibacterial Activity of the Medicinal Drink Horchata: A Study Based on the Antibacterial and Chemical Analysis of 21 Plants
Source: Molecules. 2023 Jan 10;28(2):693. doi: 10.3390/molecules28020693 (PMC9862262; doi:10.3390/molecules28020693)
Supplement: Supplementary file 1 [file molecules-28-00693-s001.zip › molecules-2057837-supplementary.pdf]

Supplementary Materials

Supplementary Table S1. Biofilm inhibition of bacterial strains of clinical importance by plant extracts used in the herbal infusion horchata.

Plant scientific name	Bacterial strains	Type	MIC	Biofilm formation (% ± SD)	*p*-values	MIC	Biofilm formation (% ± SD)	*p*-values
***Cinnamomum* sp.**	*S. aureus*	Methanol Control	1xMIC	79.5 ± 2.6	**0.002** [a]	2xMIC	80.0 ± 2.8	**0.002** [a]
		Sample	1xMIC	91.9 ± 7.2	**0.023** [b]	2xMIC	96.2 ± 7.8	**0.018** [b]
	MRSA 333	Methanol Control	1xMIC	75.2 ± 1.5	**0.003** [a]	2xMIC	80.5 ± 7.0	**0.002** [a]
		Sample	1xMIC	80.7 ± 3.4	0.088 [b]	2xMIC	85.3 ± 3.5	0.130 [b]
Pelargonium odoratissimun	*S. aureus*	Methanol Control	1xMIC	83.7 ± 9.8	**0.024** [a]	2xMIC	77.2 ± 3.2	**0.002** [a]
		Sample	1xMIC	92.1 ± 12.1	0.219 [b]	2xMIC	96.8 ± 21.7	**0.018** [b]

MIC: Minimum inhibitory concentration. SD: Standard deviation. Biofilm formation values of methanol/water controls and samples were calculated as the percentage of pathogen biofilm formation through the optical density comparison between methanol controls/samples and pathogen growth in only medium culture (positive control). The experimental positive controls were considered as 100 % when compared to methanol control and samples in the assays. Statistical analyses (*p*-values) were performed using a non-parametric Mann-Whitney test (95% confidence interval) for comparison between biofilm formation values. [a] *p*-values obtained when comparing positive controls and methanol controls. [b] *p*-values obtained when comparing methanol controls and samples. Bold *p*-values illustrated statistically significant values below 0.05.

Supplementary Table S2. Biofilm eradication of bacterial strains of clinical importance by plant extracts used in the herbal infusion horchata.

Plant scientific name	Bacterial strains	Type	MIC	Biofilm formation (% ± SD)	*p*-values	MIC	Biofilm formation (% ± SD)	*p*-values
***Cinnamomum* sp.**	*S. aureus*	Methanol Control	1xMIC	79.2 ± 7.5	**0.013** [a]	2xMIC	86.0 ± 10.8	0.068 [a]
		Sample	1xMIC	96.6 ± 19.9	0.109 [b]	2xMIC	98.3 ± 11.7	0.163 [b]
	MRSA 333	Methanol Control	1xMIC	86.4 ± 17.2	0.092 [a]	2xMIC	74.5 ± 9.3	**0.002** [a]
		Sample	1xMIC	95.9 ± 9.9	0.454 [b]	2xMIC	100.8 ± 19.2	**0.038** [b]
Pelargonium odoratissimun	*S. aureus*	Methanol Control	1xMIC	80.9 ± 10.8	**0.023** [a]	2xMIC	87.7 ± 7.4	0.109 [a]
		Sample	1xMIC	100 ± 23.9	0.283 [b]	2xMIC	100.0 ± 24.5	0.334 [b]

MIC: Minimum inhibitory concentration. SD: Standard deviation. Biofilm formation values of methanol/water controls and samples were calculated as the percentage of pathogen biofilm formation through the optical density comparison between methanol controls/samples and pathogen growth media culture only (positive control). The experimental positive controls were considered as 100 % when compared to methanol control and samples in the assays. Statistical analyses (*p*-values) were performed using a non-parametric Mann-Whitney test (95% confidence interval) for comparison between biofilm formation values. [a] *p*-values obtained when comparing positive controls and methanol controls. [b] *p*-values obtained when comparing methanol controls and samples. Bold *p*-values illustrated statistically significant values below 0.05.
